# Opioids in Cancer Development, Progression and Metastasis: Focus on Colorectal Cancer

**DOI:** 10.1007/s11864-019-0699-1

**Published:** 2020-01-22

**Authors:** Adrian Szczepaniak, Jakub Fichna, Marta Zielińska

**Affiliations:** Department of Biochemistry, Faculty of Medicine, Medical University of Lodz, Lodz, Poland

**Keywords:** Cancer, Colorectal cancer, Endogenous opioid system, Opioid receptors, Opioids, Opium

## Abstract

So far, opioids have been successfully used to reduce cancer pain in patients in order to improve their quality of life. However, the use of opioids leads to numerous side effects such as constipation, drowsiness, nausea, itching, increased sweating and hormonal changes. In this review, we described the action of opioids in several molecular pathways significant for maintenance of the intestinal homeostasis including the impact on the intestinal epithelium integrity, changes in microbiome composition, modulation of the immune system or induction of apoptosis and inhibition of angiogenesis. We summed up the role of individual opioids in the processes involved in the growth and development of cancer and elucidated if targeting opioid receptors may constitute novel therapeutic option in colon cancer.

## Introduction

Colorectal cancer (CRC) is the third most commonly diagnosed cancer in men and the second in women, what makes it an increasingly serious global problem [1•]. Inherited susceptibility is a prominent predictor of CRC development [2]. Studies confirm that certain dietary factors, namely the high intake of red meat and animal fat, as well as highly processed products can promote CRC [3]. Currently, CRC treatment is mainly based on surgical removal of cancer tissues, chemotherapy and radiotherapy. In addition to the standard immunotherapy, cellular treatment or gene therapy can also be used [4].

Opioids are commonly effective painkillers, recently tested as anticancer agents. These compounds act on the endogenous opioid system, which consists of four receptors coupled with protein G (mu, delta, kappa and nociceptin) and four major peptide families (β-endorphin, enkephalin, dynorphin and nociceptin/orphanin FQ) [5]. Opioid receptors are found mainly in the central and peripheral nervous system but also in peripheral tissues. Interestingly, the expression of opioid receptors was also found in various cancer cells such as breast, hormone-dependent (MCF-7) and hormone-independent (MDA-MB-231), colon (HT-29 and HCT116), lung (A549), bladder (MGH-U1) or neuronal (SH-SY5Y) [6].

**Fig. 1:**
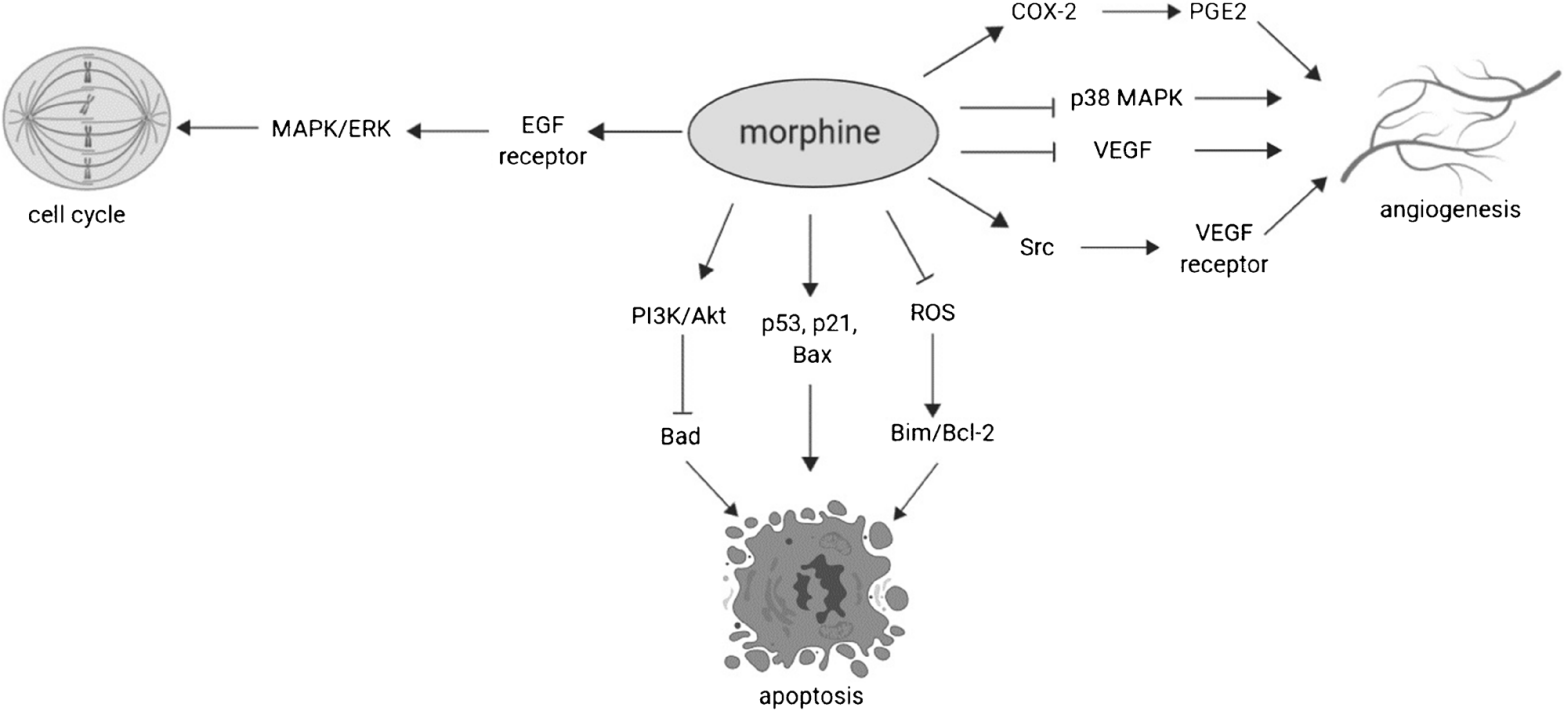
Mechanism of action of morphine on the tumor cell, modulating cell cycle, apoptosis and angiogenesis

Numerous preclinical studies indicate that opioids can play a role in the maintenance of the homeostasis of the gastrointestinal (GI) tract; moreover, it was proved that opioid ligands, both agonists and antagonists, may influence progression or inhibition of cancer growth (Fig. 1) [7•, 8].

It should be noted that opioids may increase the risk of cancer. For example, the consumption of opium and its derivatives resulted in a 4-fold increase in the risk of the upper GI tract cancer in Iran Case-control study [9]. Other studies confirmed that opiates constitute a risk factor for tumor development in the stomach [10], oral cavity [11] and esophagus [12]. Frenklakh et al. [13] and Harari et al. [14] reported that chronic opioid consumption causes significant pathological changes in the small intestine and colon. Other epidemiological studies have found that there is a link between opium dependence and initiation of GI cancers [10, 12]. In opposition to the quoted results, it was also found that opium does not promote cancer initiation of GI cancer. Alzaidi et al. [15] found that opium did not induce histopathological aggravation such as hyperemia of central veins, inflammation occurring in rodent model of GI cancer [15, 16].

## Opioids influence intestinal epithelium integrity

**Fig. 2:**
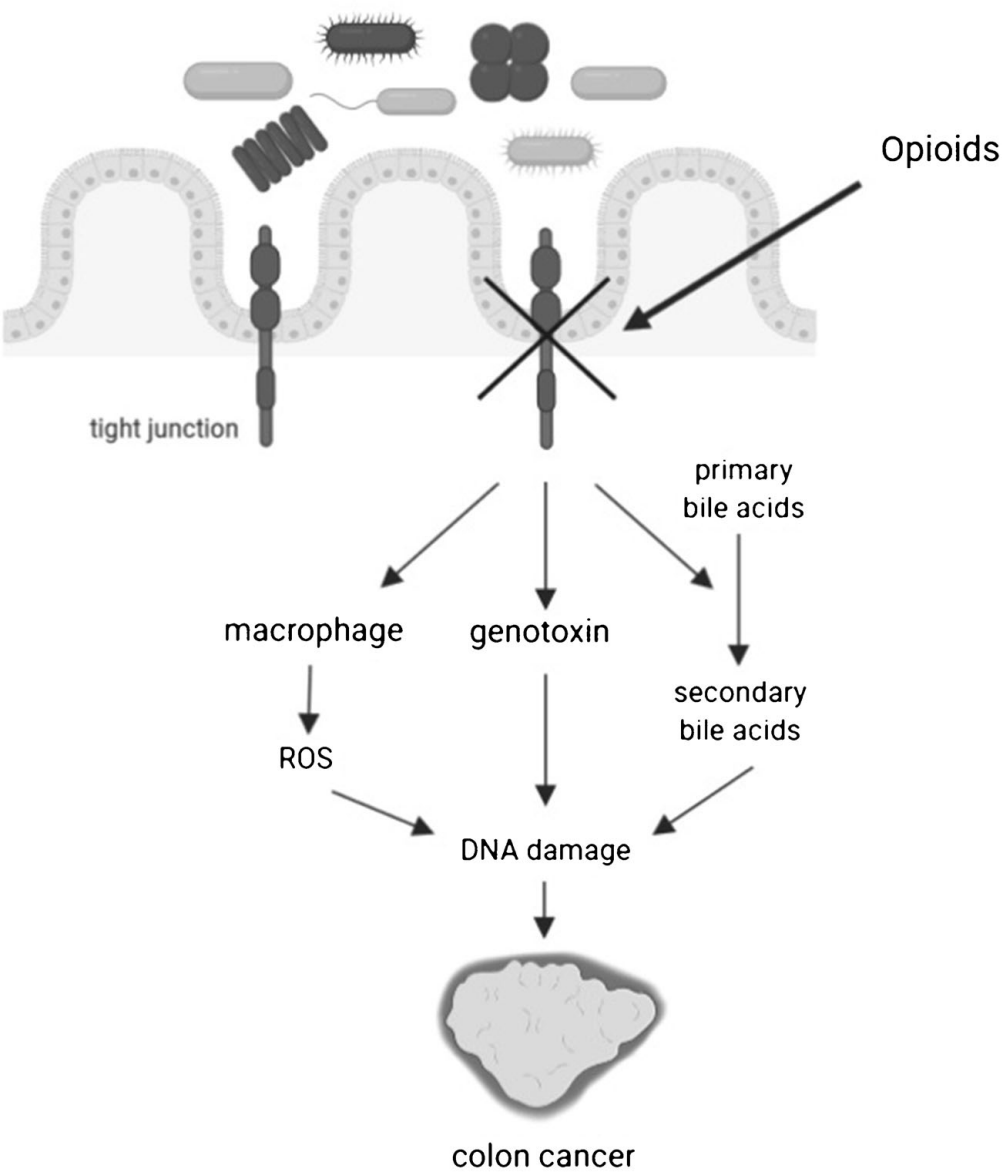
Disturbing the integrity of the epithelium by modifying tight junction has serious consequences, causing translocation of bacteria, leading to the activation of macrophages, genotoxic changes that are the causes of colon cancer

The tight junction (TJ) proteins, including occludin, claudins and zonula occludens (ZO) are essential to maintain the integrity of the epithelial barrier [17•, 18]. It is worth noting that epithelial barrier damage may contribute to the development of CRC (Fig. 2).

Currently, research focuses on the indication of the connection of opioids, mainly morphine with the integrity and permeability of the epithelium. The authors showed that treatment with morphine resulted in a significant decrease in the expression of ZO-1 and occludin genes using the brain epithelium model [19].

Meng et al. [20] using *Toll-like receptor 4* (TLR4) knockout mice showed the involvement of morphine and TLR4signaling in the intestinal epithelium permeability. Morphine concentration was maintained at range of 0.6–2.0 μg/ml. In wild type mice (WT) exposed to morphine, the authors demonstrated a significant translocation of bacteria to the mesenteric lymph nodes and liver. Bacterial translocation was significantly impaired in TLR 2 and TLR4 knockout mice. In addition, significant changes in TJ proteins in the ileum were observed in WT animals exposed to morphine. In contrast, mice treated with morphine showed a disturbed localization of occludin, which suggested impaired protein recruitment to the cell membrane. As in the case of occludin, the paracellular TJ protein ZO-1 was characterized by abnormal organization after 24 h of morphine treatment. Morphine did not change the expression of occludin or ZO-1, suggesting that morphine modulated protein distribution, resulting in impaired intestinal permeability [20].

Morphine affected intestinal integrity and function not only directly by TJ modulation but also by influencing enteric glial cells (EGCs) [21••]. EGCs regulate GI function by producing Glial Derived Neurotrophic Factor (GDNF). The research was carried out on two cell lines: IEC-6 cells (rat small intestinal epithelial cells) and EGCs (rat enteric glial cells). After stimulating EGCs with morphine, an increased permeability of FITC-dextran was noted, indicating a loss of barrier integrity. It was found that morphine reduced the protective effects of EGCs. Morphine-treated EGCs (1 μM) have reduced GDNF mRNA expression by about 50% [21••].

Okura et al. [22] conducted a study to determine the effects of repeated morphine treatment on the intestinal absorption and transepithelial transport. To determine the opioid absorption, they performed analysis by an in situ loop method and checked permeability of the human cancer intestinal epithelial cells (Caco-2). Morphine absorption from the jejunum was significantly reduced (53%) in rats treated with morphine, while duodenal and ileal absorption decreased by 38 and 17%, respectively, but these changes were not significant. Additionally, after repeated treatment with 10 μM morphine for 21 days, it was demonstrated that the permeability of the opioid was twofold greater in the basolateral to apical direction than in the apical to basolateral direction. The decrease in absorption may be related, at least in part, to the stimulation of P-glycoprotein-mediated efflux [22].

## Opioids affect gut microbiome composition

The changes in the number of specific bacterial strains contribute to CRC development [18]. The occurrence of environmental changes caused by neoplastic formation may contribute to the uncontrolled growth of *Enterococcus faecalis* [23••], thus increasing the possibility of new mutations that can modify virulence, becoming a potentially harmful factor for epithelium [24]. In addition, *E. faecalis* was found to be involved in morphine-induced intestinal dysbiosis. The infection of animals treated with morphine by *E. faecalis* may result in chronic inflammation [24]. Huycke et al. [25] proved that the reactive oxygen species produced by *E.faecalis*led to DNA damage in the colon. In addition, the use of morphine contributed to the increase in *Flavobacterium* abundance in mice [23••]. *Fusobacterium* bacteria also play role in colitis and CRC development [26]. *Fusobacterium* growth was more prominent in cancer tissue compared to the adjacent normal tissue [27].

The treatment with morphine caused reduction of primary and secondary bile acids in the intestine, which is associated with the growth of pathogenic bacteria, such as *E. faecalis*. Biliary acids have been shown to mediate host resistance to *Clostridium difficile* infection. This is in line with observations that show that the transition to a high-fat diet in mice is characterized by an increase in *Erysipelotrichaceae* and is associated with an increased risk of infectious diseases and inflammation [28, 29].

## Opioids and immune system

Opioids can prevent inflammation and inhibit tumor growth, but they can also allow the cancer to escape from the suppressing action of the immune system [30]. Low doses or short term use of opioids can have a positive effect on the immune system, while long term use or high doses of opioids can induce the contrary [30]. Several types of opioids have immunosuppressive effects via MOR and thus affect all immune cells [31].

Studies conducted on a group of patients meeting the criteria for opiate dependence in whom methadone maintenance treatment (MMT) was applied for over 1 month showed its effect on the immune system. Long-term use of MMT or a higher dose of methadone may increase pro-inflammatory cytokines, i.e. IL-1β, IL-6 and IL-8. IL-1β, was correlated with the duration of MMT and TNF-α and IL-6 in plasma were correlated with the daily dose of methadone [32]. Moreover, methadone induced anti-inflammatory action in experimental colitis. Reduction in TNF-α and IL-1β was observed in rodents with colitis after central and peripheral administration of methadone [33•].

Du et al. [34] confirmed that morphine can reduce both T lymphocyte proliferation. During the study, the analgesic effect and function in immunological modulation of homogeneous opioid peptide (source: rat β-EP) and morphine of plant origin were tested. Treatment with morphine decreased the growth rate of spleen T cells and the content of T cell subtypes (CD3+, CD4+ and CD8+), while administration of β-EP had a reverse effect on the abovementioned cell populations, as well as on cytotoxicity of natural killer (NK) cells. It was found that morphine and β-EP had no effect on plasma IL-2 levels [34].

Naltrexone hydrochloride is an opioid antagonist that inhibits MOR and DOR [35, 36]. Yi et al. [37] demonstrated that low-dose naltrexone (LDN) increases the expression of opioid receptors. LDN can significantly increase CD64 expression and decrease CD206 expression. As a result of activation, macrophages release higher amounts of IL-12, TNF-α, IL-6 and IL-1β and reduced IL-10 levels, leading to inflammation or cell death [37].

Further studies showed that opioids can affect dendritic cells (DCs) [38]. In addition, Met-enk can affect DCs, significantly promote their maturation and improve their functions. Met-enk at physiological concentrations has a positive effect on the immune system, affecting various types of immune cells, such as increasing the interaction between DCs and CD4+ Th1 cells, inducing differentiation/maturation of these cells and promoting antigen presentation [39].

Fentanyl is an opioid MOR agonist, which is widely used in clinical practice for anesthesia and sedation. Beilin et al. [40] evaluated in vivo the effect of fentanyl on cytotoxicity of NK cells (NKCC). The study was conducted in 40 different patients who were exposed to a high (75–100 μg/kg) or low (1–5 μg/kg) dose of the drug during the perioperative period. Patients exposed to low doses showed faster elimination of NKCC suppression, while patients receiving higher doses of fentanyl continued to show suppression 48 h after the procedure [40].Yeager et al. [41] carried out research to determine the effect of fentanyl on congenital and acquired immunity in humans. They administered fentanyl to seven healthy people at an initial dose of 3 μg/kg, followed by an infusion of 1.2 μg/kg per h for 2 h. Short-term exposure to fentanyl resulted in a significant increase in circulating CD16+ and CD8+ lymphocytes and an increase in the population of NK cells [41].

Since the negative effects of morphine and other opioids on the immune system are well known, some researchers claim that morphine may promote tumor growth as a result of immunosuppression [42]. Indeed, many studies have shown that opioids, especially morphine and its derivatives, are highly immunosuppressive. For example, morphine influenced the synthesis and secretion of IL-2 by lymphocytes, which increased significantly after four weeks of treatment with morphine in patients with cancer pain [43]. On the other hand, long-term use of opioids in patients with cancer did not show any significant changes in the levels of any cytokine after eight days of morphine treatment [44]. Suzuki et al. [45] executed a retrospective study of the correlation between the administration of morphine oroxycodone and the development of infections in cancer patients. Authors included 60 patients who received morphine and 74 patients receiving oxycodone; 18 and 10 cases of infection were detected, respectively [45]. Other opioids, for example fentanyl, also possess immunosuppressive activity. Fentanyl was 1000× more potent than morphine to inhibit TNF-α release in mice [46]. It should be noted that the results of the study have shown that doses of fentanyl, which are clearly capable of lowering NK activity, negatively affect the development of experimental cancer metastases [47, 48].

## Opioids impact cell cycle and apoptosis

The key mechanism commonly involved in the development of cancer is the inhibition of p53 [49]. Bax (proapoptotic) and Bcl-2 (antiapoptotic) are the main proteins involved in the regulation of apoptosis [50]. Tian et al. [51] using A549 cell line assessed the influence of opioids on apoptosis. Morphine and oxycodone contributed to increased expression of p53 and Bax mRNA and reduced expression level of Bcl-2 mRNA in A549 cells, confirming that they can promote apoptosis [51].

Xu et al. [52] also revealed the effect of opioids and anesthesia on apoptosis by performing tests using serum from patients undergoing colon cancer surgery. The patients were given two types of anesthesia: propofol with concomitant thoracic epidural (PEA) or sevoflurane with opioid analgesia (SGA). Consequently, the LoVo colon cancer cells were cultured with patient serum from both groups. Cell proliferation was significantly reduced when the cells were treated with 10% PEA serum. In contrast, apoptosis of LoVo cells was significantly reduced when treated with serum from the SGA group at a concentration of 10% [52].

Tegeder et al. [53] studied the effect of morphine on the cell cycle. They reported that morphine inhibited tumor cell proliferation at concentrations of ≥ 10 μM. It was found that naloxone (Nx) caused phase G1 inhibition in HT-29 cells. The growth-inhibitory effects of morphine were increased by Nx, suggesting that the combination of morphine and Nx might be useful to supplement cancer therapy. Furthermore, the same combination caused a significant growth of cells in the G1 phase in the HT-29 cell line. The inhibition of effector caspase 3 caused almost complete inhibition of morphine induced apoptosis. The inhibition of caspase-8 caused 50–70% inhibition of apoptosis in the HT-29 cell line. In turn, inhibition of caspase-9 induced apoptosis decreased morphine-induced apoptosis by 30–50% [53].

Zagon et al. [54] investigated the effect of – [D-Ala^2^, *N*-MePhe^4^, Gly-ol]-enkephalin, morphine or etorphine at 10 μM on the apoptosis and necrosis in HT-29 cell line. The exposure to opioids led to significantly increased the number of apoptotic and necrotic cells in the supernatant collected from HT-29 cells [54]. Moreover, fentanyl reduces the Bax gene expression (proapoptotic effect) as well as the Bcl-2 gene (anti-apoptotic effect), which indicates that fentanyl has a dual effect on cancer cell apoptosis [55].

The latest study has shown that tramadol, synthetic codeine analogue, has an apoptotic inducing effect in Colo320 colorectal cancer stem cells. Tramadol increased caspase-3 activity, reduced Bcl-2 and Ki-67 immunoreactivity in Colo320 CD133(+) cells. In addition, reduced expression of Ki-67 and Bcl-2 in HCT116 CD133 (+) and CD133 (−) cells. However, the authors showed that tramadol did not affect VEGF expression in HCT116 CD133(+) and CD133(−) cells, whereas, in the case of Colo320 and Colo741 CD133(+), it was increased compared with the control group [56]**.**

## Opioids have an impact on cancer proliferation, invasiveness and progression

The key element underlying the pathogenesis and progression of cancer is abnormal activation of signal pathways within cells [57]. One of these important signal pathways is combined with NF-κB activation [58]. NF-κB induces the expression of anti-apoptotic genes such as caspase-8 inhibitor members of the Bcl-2 family [59]. Given that NF-κB has such an important role in cancer cells, targeting of NF-κB as an anticancer therapy has also been studied in combination with opioids.

In neuronal cells, morphine inhibits NF-κB through two different pathways. First, it activates the AP-1 transcription factor, causing induction of the expression of I-κB or in a second way, activating the deubiquitinating enzyme specific for ubiquitin 15 [60]. Other studies have shown that morphine activates molecular pathways combined with NF-κB [60, 61]. For example, morphine (1 μM) inhibited TNFα-induced activation of NF-κB by significant upward regulation of IκB in neuroblastoma cells [62]. Morphine (50 μM) prevented LPS-induced NF-κB nuclear binding in monocytes and neutrophils [63]. The inhibitory effect of chronic use of morphine on NF-κB has been proven. Long-term morphine treatment reduces MOR expression in HL-60 cells exposed to TPA compared to the same cells but not exposed to morphine. It has been indicated that inhibition of AP-1 and NF-κB binding may contribute to reduction of MOR level as a result of prolonged exposure to morphine [64].

The expression of cyclooxygenase-2(COX-2) and prostaglandin E2 (PGE2) is also increased in cancer [65]. Capasso et al. [66] conducted a study focusing on bidirectional action of COX-2 and morphine. The ability of meloxicam (selective COX-2 inhibitor) to decrease morphine withdrawal and the ability of morphine to induce expression of the COX-2 have been confirmed. In addition, the ability to produce PGE2 by LPS stimulated macrophages was examined using murine macrophages J774. Macrophages treated with LPS and morphine (100 μM) produced significantly higher amounts of PGE2 compared with treatment with LPS alone, so it seems that morphine administration may lead to increased expression of COX-2 [66].

The class of small non-coding RNAs referred to as microRNAs (miRNAs) have been identified as important factors in cancer [67]. The level of miR-182 is elevated in ovarian or melanoma cancer, increasing their growth and metastases [68–70]. Taking into account these observations, it can be concluded that miR-182 may play an important role in cancer formation and metastasis and induce different effects depending on the stage of cancer progression [71, 72]. Studies have shown that fentanyl inhibits colorectal cancer in mice and HCT116 cell invasion by reducing the expression of β-catenin and miR-182 [73]. It should be emphasized that miR-182 was reduced in HCT116 cells treated with 2 ng/ml fentanyl. Interestingly, fentanyl at a concentration of 2 ng/ml reduced cell invasion to about 62% [74]. Additionally, it has been identified that miR-302b mediates the effects of fentanyl on the cancer cells. Based on the results obtained, the authors found that fentanyl inhibited the proliferation and invasion of esophageal squamous cell carcinoma by increasing expression of miR-302b [75].

## Angiogenesis end metastasis

### Angiogenesis

The balance between angiogenesis activators and inhibitors is essential to maintain homeostasis in the body. Blebea et al. [76] used chick chorioallantoic membrane as an in vivo model to study angiogenesis. Three-millimeter methylcellulose disks were placed on the surface of the chorioallantoic membrane and each one of them contained: [Met5]-enkephalin(5 μg), Nx (5 μg), opioid growth factor and Nx together (5 μg of each), the long-acting opioid antagonist naltrexone (5 μg), or distilled water (control). The results showed that the opioid growth factor had an inhibitory effect on angiogenesis, both on the number of blood vessels (by 35%) and on the decrease in total vessel length (by 20%) compared with the control levels [76]. Moreover, research using fertilized sanhuang chicken eggs showed that Deltorphin I by stimulating DOR or Endomorphin-1 and -2 through MOR may increase the formation of blood vessels [77]. Based on current results, it is speculated that constitutive MOR activation is coupled with anti-angiogenic activity.

An important signaling element in the formation of new blood vessels is *vascular endothelial growth factor* (VEGF) [78•]. It has been shown that morphine stimulates the VEGF receptor through MOR activation [79]. Administration of morphine (0.5 mg/kg per day for 2 weeks) increases angiogenesis. It was also found that the administration of opioid caused an increase in IL-6, substance P and activation of mast cells. In addition, mice exposed to morphine showed higher concentrations of GM-CSF, RANTES and IL-6 in tumors compared with the control group. Additionally, it is suggested that morphine stimulates recruitment, differentiation and degranulation of mast cells [80]. Koodie et al. [81] studied the effect of morphine on angiogenesis in mice with subcutaneous injection of Lewis lung cancer cells (LLCs). A continuous slow-release of morphine pellet, maintaining morphine plasma levels within 250–400 ng/ml, caused an inhibition of angiogenesis as compared with placebo. In addition, morphine significantly reduced the density of blood vessels, vessel branching and length [81].

One of the important studies on the influence of opioids on cancer progression and angiogenesis was carried out by Gupta et al. [82] who used animal model of breast cancer. Animals received injections of morphine sulphate at the dose of 0.714 mg/kg mice per day for the first 15 days, and then 1.43 mg/kg mice per day until the 38th day. It was proved that morphine administered at clinically significant doses increased cancer progression, but also higher number of vessels, increased total length of vessels and more vascular branches in the morphine group compared to control [82].

Martin et al. [83] suggested that morphine plays both a direct and indirect role in suppressing angiogenesis during the wound healing process. They demonstrated that morphine has a direct effect on wound repair by suppressing angiogenesis in matrigel plugs containing LPS (1 μM) and heparin (20 U/L). Matrigels were injected into the hind limb of placebo or morphine pelleted mice. Gross morphology showed a marked presence of new blood vessels forming throughout the plugs of placebo treated mice. Additionally, the authors pre-incubated the macrophage cell line RAW 264 cells with morphine. They showed that at a concentration of 100 nM and 1 μM, morphine attenuated LPS-induced VEGF expression, however, in the absence of LPS, significantly reduced VEGF expression as compared with control group.

Oxycodone is an opioid analgesic, which is usually used to relieve moderate to severe pain, improving the quality of life [84]. Tian et al. showed that oxycodone affects the process of angiogenesis by reducing the level of VEGF. It was shown that, at concentration 40 μg/ml, oxycodone decreased VEGF expression levels in A549 cells. Oxycodone, in contrast to morphine, decreased the expression levels of uPA and ICAM-1 in A549 cells. These results suggest that oxycodone may be a better opioid drug candidate as an anti-cancer agent than morphine [51].

### Metastasis

Cancer metastasis is a multistage process causing the detachment of malignant cells from the primary tumor, invasion of surrounding tissues, adhesion to endothelial cells in distant places and extravasation through the endothelial membrane in order to colonize new tissues [85].

Urokinase plasminogen activator (uPA) and matrix metalloproteinases (MMPs), especially MMP-2 and MMP-9, participate in the degradation of the extracellular matrix and are therefore closely related to the occurrence and development of cancer [86, 87]. The use of morphine (10, 50 and 100 ng/ml) on metastatic colon 26-L5 carcinoma resulted in decreased production of MMP-2 and MMP-9 by these cells. In this case, morphine significantly inhibited the invasion, migration and adhesion of tumor cells without affecting the in vitro growth [88]. Moreover, the researchers have confirmed that morphine caused significantly increased uPa secretion, suggesting that this compound may increase the elasticity of cancer cells. Morphine at 0.1 μM was efficient in increasing the secretion of uPa. Current results suggest that morphine treatment can accelerate cancer progression by increasing the metastatic capacity of cancer cells [89].

Interestingly, morphine affects the production of MMP-9 by various signaling pathways, also depending on the time of exposure and the dose. Chronic or high dose of morphine leads to activation of adenyl cyclase (AC), increase in the level of cyclic adenosine monophosphate (cAMP)/protein kinase A (PKA)/CREB and consequently inhibition of NF-κB and MMP-9 production. Moreover, it has been confirmed that continuous administration of morphine at high doses can modulate the production of MMP-9, which, in consequence, inhibits tumor growth and metastasis. However, acute or low-dose morphine treatment results in activation of the opioid receptor after the release of the Gbeta-gamma complex (Gβγ) and signaling of phosphoinositide 3-kinases (PI3K)/protein kinase B (GDP), which results in activation of NF-κB and production of MMP-9 [90•]. In addition to morphine, fentanyl (2 ng/ml) downregulated the expression of β-catenin as well as MMP-9 protein in HCT116 cells [86]. Reduced expression of MMP-9 confirms that fentanyl inhibits the invasion of human colorectal cancer cells [91].

### Opioids and clinical data

Recent retrospective studies have shown that perioperative use of fentanyl is associated with reduced overall survival (OS) and increased tumor recurrence in patients with early non small cell lung cancer (NSCLC) [92]. In another study, NSCLC patients were divided into groups for oral morphine equivalents/day (< 60 or ≥ 60 mg). The results of the analyses showed that the opioid dose does not shorten the survival of patients with advanced cancer [93]**.** Janku et al. analyzed data about effects of methylnaltrexone (MNTX) on patient survival. In patients with advanced terminal tumors and opioid-induced constipation, MNTX treatment was associated with prolonged OS compared with patients treated with placebo or no response. The analysis showed that patients treated with MNTX had almost three times higher OS than patients receiving placebo (*p* < 0.001) [94]. However, Sathornviriyapong et al. determined whether there is a link between the different doses of opioids and the survival of palliative cancer patients. The retrospective study included 317 cancer patients. They showed that different doses of opioids were not associated with a shortened survival period (*p* = 0.52) [95].

Perioperative factors, including the use of general anesthesia such as volatile anesthesia or opioids, may impair host defense against tumor recurrence. However, the use of regional anesthesia-analgesia may bypass adverse effects. Sessler et al. conducted a randomized controlled trial in which they assumed that the recurrence of breast cancer after a potentially cured surgery is lower under regional anesthesia using paravertebral blocks and anesthetic propofol than under general anesthesia. They showed that local anesthesia did not reduce the recurrence of breast cancer after surgery compared with volatile anesthesia (sevoflurane) and opioids. This study clearly indicates that general or local anesthesia can be equally valuable for clinicians in terms of its impact on cancer [96].

In conclusion, there are no clear results indicating that opioids used in the perioperative period in patients undergoing cancer surgery promote recurrence of cancer and metastasis. Currently, it is not justified to support opiophobia, while opioids remain indispensable in the treatment of pain in cancer patients [97].

### Interactions between opioids and chemotherapy

The use of opioids may modulate the activity of chemotherapists in cancer treatment. Patients are often treated with analgesics and anticancer drugs, but the influence of these drugs on each other is not fully understood. It was shown that morphine modulates cisplatin-induced apoptosis in human CNE-2 cells of nasopharyngeal cancer and thus affects the anticancer activity of cisplatin. Treatment with morphine (1 μg/ml) inhibited cisplatin-induced apoptosis of CNE-2 cells, increasing the Bcl-2/Bax ratio. However, the effect is dose-dependent because a high dose of morphine (1000 μg/ml) had the opposite effect. In addition, at a low dose, morphine increases the chemo-resistance in the model of nosopharyngeal cancer in vivo by inhibiting cisplatin-induced apoptosis [98]. Another study confirmed that tramadol and flurbiprofen inhibit cisplatin cytotoxicity by inhibiting the activity of fissure joints, but morphine did not show this effect. The inhibition of junctional communication may counteract the action of anticancer agents [99].

## Conclusion and future perspectives

Opioids do not only have an effect on nociception but also on cell proliferation, cell death and immune system; their application may change the function of cancer cells. The currently available results revealed contradictory conclusions, indicating both positive (opioids through activation of the immune system lead to increased production of pro-inflammatory cytokines) and negative (opioids may induce apoptosis of cancer cells) effects on the cancer development and metastasis. For example, morphine and fentanyl, the best known and most widely used drugs, showed inhibitory effects on the growth, metastasis and proliferation of cancer cells. However, side effects induced by them limit their use as anti-cancer agents. Finally, it needs to be underlined that the results of the research on opioids’ role in cancer development and metastasis are dependent on the used model, the type of cancer, the doses of opioids, route of administration or the time and method of exposure to opioids. So far, it is really difficult to conclude if the use of opioids may constitute a new option in anti-cancer therapy.

So far, studies have shown that opioids have anti-cancer properties and are a promising therapeutic strategy. However, in the future, it is important to focus on some key aspects in the field of opioids. One of the main objectives should be to thoroughly examine the influence of opioids on particular types of cancer. In order to obtain effective treatment, it is necessary to design drugs that are selective and specific in relation to cancer cells. In case of colorectal cancer, it would be advisable to construct medicines acting in selected segments of GI. Finally, opioids cause serious side effects, which should be eliminated in new opioid drugs.

## Abbreviations

COX-2: cyclooxygenase-2.
CRC: colorectal cancer.
DCs: dendritic cells.
DEN: diethylnitrosamine.
DOR: delta opioid receptor.
EGCs: enteric glial cells.
EMT: epithelial-mesenchymal transition.
GDNF: Glial Derived Neurotrophic Factor.
GPCR: Gprotein-coupled receptors.
IBD: inflammatory bowel disease.
IL-1β: *interleukin* 1β.
IL-6: *interleukin 6.*
IL-8: *interleukin 8.*
KOR: kappa opioid receptor.
LDN: low-dose naltrexone.
MMPs: matrix metalloproteinases.
MMT: methadone maintenance treatment.
MOR: mu opioid receptor.
n-Cdh: n-cadherin.
NK: natural killer cell.
NKCC: natural killer cell cytotoxicity.
Nx: naloxone.
ORL1: opioid receptor-like receptor 1.
PEA: propofol with epidural anesthesia.
PGE2: prostaglandin E2.
TJ: tight junction.
TLR: toll-like receptor.
TNFα: tumor necrosis factor α.
TPA: 2-o-tetradecanoyl-phorbol-13-acetate.
uPA: urokinase plasminogen activator.
ZO: *zonula*occludens.
